# Family education in schizophrenia: A comparison of two approaches

**DOI:** 10.4103/0019-5545.43056

**Published:** 2005

**Authors:** R. Thara, R. Padmavati, A. Lakshmi, P. Karpagavalli

**Affiliations:** * Director, Schizophrenia Research Foundation (SCARF), Chennai; ** Consultant Psychiatrist, Schizophrenia Research Foundation (SCARF), Chennai; *** Case Manager, Schizophrenia Research Foundation (SCARF), Chennai; **** Case Manager, Schizophrenia Research Foundation (SCARF), Chennai

**Keywords:** Family education programme (FEP), schizophrenia, primary caregivers

## Abstract

**Background:**

Family education programme (FEP) for families of persons with schizophrenia is practised in several centres as a part of patient-and family-related services.

**Aim:**

This paper describes two models of FEP conducted at the Schizophrenia Research Foundation (SCARF), Chennai.

**Methods:**

The first programme was a part of a research study and was structured utilizing standard evaluation instruments. The second was flexible and tailored to the needs of the family members.

**Results:**

After the first programme, the psychopathology of patients and the burden of caregiving on primary caregivers did not show any significant difference but there was a significant gain in caregivers' knowledge with information and experience sharing. Most families seemed to prefer the second programme, which recorded better attendance and participation.

**Conclusion:**

Informal educational sessions with periodic ‘across-the-table’ re-inforcers may be more effective and practical in the Indian setting.

## INTRODUCTION

Family psychoeducation has established its efficacy and effectiveness as an evidence-based practice. Most mental health professionals believe that educating the family members of patients with schizophrenia on the various aspects of the illness not only helps them to cope better, but also leads to more definite improvements in the clinical status and functioning of the patients.

In a review of the rationale behind the family education programme (FEP), Goldstein^1^ observed that these were designed to prevent or delay relapse in patients with schizo-phrenia. Since most of these programmes have been oriented toward patients and their close relatives, the studies reviewed have tested the effect of these programmes when added to maintenance pharmacotherapy. The results of the first generation of studies carried out in the late 1970s and 1980s confirmed the positive effects of a family-based psycho-educational programme on delaying the recurrence of an episode of schizophrenia.^2^–^5^ More recent studies, termed as second generation, have built on these findings to test more specific hypotheses concerning the most efficient format for delivery of such programmes (relatives only groups, single family unit therapy, multiple family groups). While no clear advantage has been found for any one format, results of these studies have suggested that intensive family involvement in the community care of patients with schizophrenia and other psychotic patients may be a critical ingredient in successful relapse prevention programmes.

The Schizophrenia Research Foundation (SCARF) is a non-governmental organization engaged in the care and research on schizophrenia since 1984. SCARF has been conducting FEPs for over 10 years. This paper describes two different formats of FEPs but does not compare the two methods. Objective data and subjective reports were examined to determine the efficiency of the two modalities of conducting FEPs in the Indian setting.

During the study period of 24 months, two different formats were used for conducting FEPs; one was a structured programme with an inbuilt research component and the other a loosely knit, more informal approach. Both formats involved multifamily groups.

## PROGRAMME I: STRUCTURED PSYCHOEDUCATION PROGRAMME

### Methods

This programme was held from 1998 to 2000 and its primary objective was to evaluate the efficacy of structured psycho-education for families of patients with chronic schizophrenia.

Thirty primary caregivers of patients with schizophrenia were included in the study to assess the impact of organized family education. All families were requested to attend a six-week programme entailing six sessions of 2 hours each (Table 1). Despite rigorous persuasion, 4 caregivers dropped out in the initial stages and hence were not included in the analysis. The reasons for dropout were: one had to leave Chennai; one had illness in the family; one found it difficult to travel; and one was not convinced about the utility of the programme. Of the remaining 26 caregivers, 2 did not attend all the sessions. Eight to ten families attended all the sessions of the programme.

**Table 1:** Content of the structured programme

Session	Content
Session 1	Information about the illness
Session 2	Illness management
Session 3	Crisis management
Session 4	Coping strategies/problem-solving
Session 5	Managing stress and dealing with emotions
Session 6	Addressing frequently asked questions (FAQs)

#### Inclusion criteria

1. Only the residents of Chennai were included. This was essential to ensure regular attendance in the sessions
2. Patients who satisfied the DSM-IV criteria for schizo-phrenia were included.
3. Only those primary caregivers who had not been exposed to any formal psychoeducation programme earlier at either SCARF or any other centre were selected.

#### Tools used

The scales used for making baseline assessments in patients and caregivers are given in Box 1.

**Box 1:** Scales used for baseline assessments

For patients
Positive and Negative Syndrome Scale (PANSS) for psychopathology Schedule for the Assessment of Psychiatric Disability^6^
For caregivers
Hamilton Anxiety Scale Hamilton Depression Rating Scale Burden Assessment Scale (BASS)^7^

Final assessments were made at the end of session 6. Due to logistic reasons, interim assessments could not be made, although they were initially envisaged.

### Results

#### Patients

Sixteen men (mean age: 33.69±6.89 years) and 10 women (mean age: 33.30±4.37 years) fulfilling the DSM-IV criteria for schizophrenia were selected. The mean duration of illness was 7.5±1.2 years (range: 2–35 years).

#### Primary caregivers

All the primary caregivers were residents of Chennai, living within a distance of 1–25 km. Most were female or retired male members of the family, and were from the middle or low socioeconomic class (62.8%), reflecting the section of people accessing the services of SCARF. Of the primary caregivers, 9 were mothers, 6 fathers, 4 siblings, 6 spouses and 1 offspring. The mean age of primary caregivers was 50.96±14.07 years (range: 43–72 years).

Statistical analysis was done using paired *t* test to compare the pre- and post-session scores. Scores on all the three subscales of PANSS—positive, negative and general psychopathology—registered a decrease (Table 2). There were no statistically significant differences between the subscales. Scores on all the areas of disability, namely, personal, social and occupational, registered a decline. Significant differences were seen in the area of occupational disability (Table 3). There were no significant differences in the caregivers' scores of burden, depression and anxiety.

**Table 2:** PANSS scores of patients whose families attended the family education programme

Subscales	Pre-assessment	Post-assessment	Statistics
Positive	15.85	12.69	*t*=1.35, df=22, p=0.272
Negative	14.15	11.96	*t*=1.19, df=22, p=0.247
General	30.00	26.46	*t*=s1.21, df=22, p=0.262
psychopathology			

PANSS: Positive and Negative Syndrome Scale

**Table 3:** Disability scores of patients whose families attended the family education programme

Disability	Pre-assessment	Post-assessment	Statistics
Personal	9.11	8.69	*t*=0.55, df=22, p=0.791
Social role	7.01	4.65	*t*=1.15, df=22, p=0.212
Occupational	8.73	5.01	*t*=2.69, df=22, p=0.021*

* Significant at p<0.02

#### Observations

The information imparted during the sessions was well received. The sessions provided a forum for information sharing and interaction between families. Issues pertaining to the illness were raised and discussed. There was an increase in the knowledge about the illness as evidenced during feedback interviews with the primary caregivers, which indicated that the programme was useful in terms of knowledge gains and practical applications. However, this was only an observation and no standard tool was used to measure the increase in knowledge.

## PROGRAMME II: INFORMAL PSYCHOEDUCATION PROGRAMME

This method was regularly practised at SCARF in a clinical setting and did not involve the use of any evaluation tool. Families were recruited using the same inclusion criteria as used for the previous programme. This format consisted of a single session of 3 hours comprising the following:

- Screening of a 40-minute film called FACES was held (Family care, empowerment and support), which was produced during the structured psychoeducation study. This film showed 3 case vignettes; after each case, the common problems faced by the families (e.g. in marriage, employment, etc.) were discussed by a mental health professional.
- Interactive session of primary caregivers with professionals and among themselves.
- This was followed by the routine and regular re-inforcer sessions across the table during the reviews.

Six sessions were held over a period of 4 months. These sessions were held on Saturdays for groups of 10–12 primary caregivers, by the same investigators. No formal assessments were made since this format was not intended as a research design. However, at the end of each session the participants were asked about their observations, opinions and suggestions.

Only one primary caregiver failed to turn up for these programmes. The entire interactive session was enthusiastic and meaningful. A few primary caregivers even requested that they be invited for another similar session.

### Observations

Time seemed to be an important factor for the families attending the programme. The 3–4 hour sessions were well attended. The general consensus was that such programmes need not be very structured and should allow enough time for discussion. The primary caregivers also opined that it would be difficult for them to attend more than two such sessions.

## DISCUSSION

There is little dispute that families of patients with chronic schizophrenia benefit from information about the illness and support. This paper deals with the experience of two types of FEPs for caregivers at SCARF. In the structured programme extending over 6 weeks, the problem was to ensure regular attendance. Since this was done as a research study, the staff had to use all the persuasive and alluring techniques to ensure attendance of all the sessions in the programme. It this had not been the case, the attendance rate would have been lower.

Assessment done following the completion of the programme did not reveal significant changes in the psychopathology or burden scores. Occupational disability had improved, but a careful analysis revealed that multiple factors such as intensity of rehabilitation inputs also contributed to this finding.

However, all participants expressed a lot of satisfaction with the content of the programmes, which had resulted in gain in knowledge for them. It also enabled them to brainstorm on some issues of common interest with other families who had participated in the programme.

Multifamily psychoeducation groups allow for an increase in the size and strength of social support network, connecting families that have similar problems, providing a forum for mutual aid, sharing workable solutions and building hope through mutual examples and experiences.^8^ Participants in this study also reported that they welcomed the opportunity to share their experiences and identify possible solutions to the common problems faced.

The present study did not find any significant differences in the scores of psychopathology or burden on carers. De Groot *et al.* undertook a telephone survey for two groups of families—one group was receiving brief multiple family psychoeducation and the other was not participating in the programme—to investigate the knowledge and understanding of services and burden of care.^9^ While participation in the brief multiple psychoeducation programmes did not reduce the number or duration of admissions of patients or impact on the level of burden of care on caregivers, there was evidence of increased knowledge and understanding of services. McDonell *et al.* also observed that there was no reduction in the caregivers' burden after psychoeducation.^10^ In Finland, Stengard compared the two methods of oral and visual educational interventions.^11^ The education led to considerable gain in knowledge and increase in the psychological well-being of the participants, but there were no significant changes in objective burden or expressed emotion status after intervention. Multiple therapeutic approaches would be necessary to reduce behaviours and disruption related to schizophrenia and to ameliorate family burden and thereby enhance outcomes. The authors did not find any Indian studies on FEP in the past 10 years to make any comparisons.

The evidence suggests that brief educational intervention can yield significant gain in knowledge of the caregivers, who will then be better equipped in dealing with patients with schizophrenia. It was also observed that families actively sought information and were more receptive in a crisis. Better-designed, randomized studies are needed to prove the definite efficacy of brief educational interventions.

It is therefore not too presumptuous to conclude that an FEP may not result in striking changes in the psychopathology or disability of the patient or even in the reduction of family burden. The advantages of an FEP are an increase in knowledge, an opportunity for brainstorming and sharing, and subjective sense of well-being of families. Most families seem to prefer short sessions with periodic across-the-table re-inforcer sessions rather than well-structured, long programmes.

One limitation of the study was that the assessment of the second approach, which was not conceived as a research study, was not as rigorous as the first and did not involve the use of standardized instruments. Its efficacy was measured by the better attendance, participation and subjective views of the participants as expressed to the team at SCARF. The feedback was however convincing enough to conclude on the preference of this type of approach.

## CONCLUSION

This paper describes two types of FEPs for families of persons suffering from schizophrenia. The insignificant changes in symptoms and burden after the well-structured programme does not take away the merits of the programme which had resulted in knowledge gains and better well-being. However, these were also achieved by the second, less structured shorter programme followed by periodic re-inforcer sessions. Hence we suggest that the second may be more suitable in the Indian setting.
